# IKBKB在人肺腺癌细胞株A549及其耐药细胞株A549/DDP中的表达和意义

**DOI:** 10.3779/j.issn.1009-3419.2014.05.01

**Published:** 2014-05-20

**Authors:** 康 齐, 洋 李, 雪冰 李, 芳 张, 宜 邵, 清华 周

**Affiliations:** 300052 天津，天津市肺癌转移与肿瘤微环境重点实验室，天津市肺癌研究所，天津医科大学总医院 Tianjin Key Laboratory of Lung Cancer Metastasis and Tumor Microenvironment, Tianjin Lung Cancer Institute, Tianjin Medical University General Hospital, Tianjin 300052, China

**Keywords:** IKBKB, NF-κB, 耐药, 肺肿瘤, IKBKB, NF-κB, Drug-resistant, Lung neoplasms

## Abstract

**背景与目的:**

肺癌顺铂耐药在临床治疗中广泛存在，严重影响了肺癌患者的治疗效果，因此研究肺癌耐药机制对新药的研发和解决临床肿瘤耐药有十分重要的意义。IKBKB是组成IKK复合物重要的催化亚基之一，其对核转录因子NF-κB的激活起到重要调控作用。本研究旨在探讨*IKBKB*基因在肺腺癌顺铂耐药细胞株（A549/DDP）与其亲本肺腺癌A549细胞株中的表差异及其调控肺腺癌顺铂耐药的机制。

**方法:**

应用MTT法检测A549和A549/DDP细胞株顺铂敏感性及*IKBKB*基因对A549细胞株顺铂耐药性的影响。Real-time PCR检测肿瘤细胞中*IKBKB*基因mRNA变化，流式细胞学检测肿瘤细胞凋亡率，双荧光素酶报告基因实验检测NF-κB的活性。

**结果:**

A549细胞与A549/DDP细胞在IC_50_和凋亡率方面均有统计学差异，*IKBKB*基因在A549/DDP细胞株中mRNA表达水平明显高于A549。与对照组比较，pcDNA3.1/IKBKB转染A549细胞后，*IKBKB*基因在mRNA水平明显升高，A549细胞顺铂耐药性明显增加，IC_50_增加2.85倍，凋亡率减少59%，NF-κB的活性明显升高。

**结论:**

*IKBKB*基因通过激活NF-κB信号通路抑制细胞凋亡，从而导致细胞耐药性增加，这一研究结果对新抗肿瘤药物的研发和解决肿瘤耐药难题有重要意义。

## Abstract (en)

**Background and objective:**

Cisplatin-resistance in Lung cancer cells is widespread in the clinical treatment, seriously affecting the effects of the treatment of lung cancer. Therefore, the research of mechanisms of cisplain-resistance has significant meaning for developing new chemotherapeutic drug and solving the cisplain-resistance in clinic treatment. IKBKB is one of the most important catalytic subunits of IKK complexes. It plays an important regulatory role in activation of NF-κB. The aim of this study is to investigate the differential expression of *IKBKB* gene in human lung adenocarcinoma cells line A549 and the cisplatin-resistant variant A549/DDP and the mechanisms of cisplain-resistance induced by *IKBKB* gene.

**Methods:**

MTT assay was employed to determine the sensitivity of A549 and A549/DDP cells line to cisplatin and the effect of *IKBKB* gene on A549 cell lines' sensitivity to cisplatin. The mRNA level of IKBKB was determined by real-time PCR. Dual luciferase reporter gene experiment was employed to determine the activity of the NF-κB. Apoptosis rate of lung adenocarcinoma cells was determined by flow cytometry.

**Results:**

Apoptosis rate and IC_50_ were significantly different in A549 and A549/DDP cells, the expression of mRNA level of *IKBKB* gene in A549/DDP was significantly higher than that in A549. Compared with control group, *IKBKB* gene was able to reduce the cisplain sensitivity of A549 cells. After A549 was transfected with pcDNA3.1/IKBKB plasmid, mRNA level of IKBKB was significantly increased, the sensitivity of cisplain was decreased, the IC_50_ was increased 2.85 fold, the apoptosis rate was decreased 59%, the activity of NF-κB has been greatly increased.

**Conclusion:**

IKBKB inhibits cisplatin-induced apoptosis via the activation of NF-κB pathway. It will be helpful in the development of new anticancer drug and solving the challenge of cisplatin-resistance.

肺癌是世界上最常见的恶性肿瘤之一，其死亡率占全球所有男性肿瘤患者死亡人数的28%，女性肿瘤患者死亡人数的26%^[1]^。其中非小细胞肺癌占到了肺癌总数的80%^[2]^。化疗是肺癌综合治疗方案的重要组成部分，由于近些年化疗方案的不断改善，肺癌患者生存率已得到明显提升，但是肿瘤细胞耐药性的产生严重影响了化疗的疗效。顺铂是肺癌化疗方案中最常应用的药物之一^[3]^，但是在临床治疗过程中顺铂耐药现象广泛存在。因此，研究顺铂耐药机制对探索研发克服耐药产生的新化疗药物有重要意义。通过研究发现，顺铂耐药机制主要包括DNA损伤修复能力增强、细胞周期的调控、细胞解毒功能增强及细胞凋亡的抑制等^[4-6]^。

IKBKB（又名为IKKβ）是IKK复合物重要的催化亚基，它与催化亚基IKKα以及调节亚基IKKγ共同构成IKK复合物。IKK复合物的磷酸化可以激活NF-κB，使得NF-κB转入细胞核内调控一系列基因表达^[7, 8]^。在组成IKK复合物的催化亚基中，IKBKB在激活NF-κB方面起到最重要的作用^[9]^。最近研究^[10, 11]^发现，IKBKB在乳腺癌和头颈部鳞癌顺铂耐药产生中起重要作用。然而，IKBKB在肺癌顺铂耐药性产生的作用中罕有报道。

在本研究中，我们发现*IKBKB*基因高表达可以导致肺腺癌细胞顺铂耐药性增加并抑制细胞凋亡。实验结果显示*IKBKB*基因在肺癌顺铂耐药过程中发挥重要作用，该基因主要通过激活NF-κB信号通路抑制细胞凋亡导致耐药。

## 1 材料与方法

### 1.1 主要材料与仪器

本实验采用人肺癌腺A549癌细胞系及人肺腺癌顺铂耐药细胞系A549/DDP由天津市肺癌研究所提供；顺铂、噻唑蓝（MTT）、二甲基亚砜（DMSO）购自Sigma；细胞培养基PRMI-1640购自GIBCO；质粒小提试剂盒及胶回收试剂盒购自Axygen；质粒表达载体3.1(+)Hygro及转染试剂lipofectamine 2000购自Invitrogen；感受态细胞（E.coli DH5α Competent Cells）；限制酶（*Hind* Ⅲ, *Xba* Ⅰ）、M-MLV逆转录酶、实时荧光定量PCR试剂盒购自Takara；细胞凋亡流式检测试剂盒购自BD biosciences；PCR仪（Thermo）；流式细胞仪（BD）；酶标仪（Thermo）；质粒测序由北京华大基因公司完成。

### 1.2 细胞培养

A549及A549/DDP细胞使用含有10%小牛血清的PRMI-1640培养基至于37℃、5%CO_2_培养箱中培养，0.25%胰蛋白酶消化传代，实验均采用处于对数生长期的细胞。其中A549/DDP细胞在培养过程中置于2 μg/mL DDP中维持其耐药性。

### 1.3 质粒构建与转染

根据*IKBKB*基因CDS区设计引物序列为：上游引物：5' - AAGCTTCGACATCAGTATGAGCTGGTCACCTTCC-3' ；下游引物：5' - TCTAGAAGTCCCCCCACATCATGAGGCCTGCTCC-3' ；以人正常支气管上皮细胞RNA为模板反转录cDNA。PCR条件：98 ℃、30 s，1个循环；98℃、10 s，63℃、10 s，68℃，2 min 40 s，34个循环；68℃、2 min，4℃保存。PCR产物电泳后胶回收并测序。*IKBKB*基因胶回收产物与pcDNA3.1空载体均用*Hind* Ⅲ和*Xba* Ⅰ进行双酶切，酶切产物电泳回收，用T4DNA Ligase在16℃连接回收的IKBKB片段和pcDNA3.1载体。连接产物利用DH5α感受态进行转化，转化产物涂于含氨苄青霉素（Amp）的LB琼脂板上，37℃培养过夜。待LB琼脂板长出菌落后，挑取单菌落至于液体LB培养基中摇菌扩增。应用质粒小提试剂盒提取质粒并进行酶切及测序验证。

细胞转染：将A549细胞接种到6孔板中，细胞生长达到80%融合时，根据lipofectamine 2000说明书进行转染。

### 1.4 MTT检测细胞耐药

将培养皿中对数生长且融合度达到80%的A549细胞、A549/DDP细胞和转染pcDNA3.1/IKBKB质粒的A549细胞及转染空载体pcDNA3.1质粒的A549细胞用胰酶消化，培养基终止消化后制成1×10^4^/mL单细胞悬液，加入到96孔板中，每孔180 μL。在37 ℃、5%CO_2_培养箱中培养24 h后，将顺铂稀释为0、0.5 μg/mL、1 μg/mL、2 μg/mL、4 μg/mL、6 μg/mL、8 μg/mL、10 μg/mL、12 μg/mL、16 μg/mL、32 μg/mL的浓度梯度，每一浓度梯度设置5个附孔，并且设置空白对照空（仅有细胞）及空白孔（仅有培养基），72 h后每孔加入20 μL新制备的MTT（5 mg/mL）溶液，在孵箱中培养4 h后，吸去培养基，每孔加入150 μL DMSO溶液，微量振荡器震动10 min，应用490 nm波长的酶标仪（Molecular Device SPECTRA max M5, USA）测定96孔板吸光值（OD）。抑制率计算公式为：生长抑制率=（1-OD_用药组_/OD_对照组_）×100%，以最小二乘法进行曲线拟合，得到IC_50_值。

### 1.5 Real-time

PCR检测A549、A549/DDP、转染pcDNA3.1/IKBKB质粒的A549细胞、转染空载体pcDNA3.1质粒的A549细胞中的mRNA水平变化，以及进一步检测*NF-κB*基因mRNA水平变化取对数生长的上述细胞，用Trizol提取各细胞系的总RNA，IKBKB上游引物: 5' -GGGAAATAACACCCGAAAG-3' ；下游引物：5' -TGTAACTGAACATAAAC-3' ；GAPDH上游引物：5' -TGCACCACCAACTGCTTAGC-3' ；下游引物：5' -GGCATGGACTGTGGTCATGAG-3' ；反应条件：95℃、30 s，一个循环；95℃、5 s，60℃、30 s，40个循环；95℃、15 s，60℃、1 min，95℃、15 s，一个循环。

### 1.6 流式细胞技术

检测*IKBKB*基因在肺腺癌细胞耐药中的作用，将对数生长的A549细胞制备成1×10^4^/mL单细胞悬液，加入到6孔板中，每孔2 mL。在37 ℃、5%CO_2_培养箱中培养12 h，根据lipofectamine 2000说明书转染pcDNA3.1/IKBKB质粒及pcDNA3.1空载体（空载体组为对照组）。转染后36 h后，每孔加入2.5 μg/mL浓度的顺铂，继续孵育24 h。细胞用胰酶消化后根据说明书应用Annexin V-FITC凋亡检测试剂盒染色（Annexin V-FITC Apoptosis Analysis Kit, Pharmingen, CA），用FACSAria™流式细胞仪（BD Bioscience, CA）检测染色细胞，以转染pcDNA3.1空载体的A549细胞为对照组，WIMDI 2.8软件进行分析。

### 1.7 双荧光素酶报告基因实验

从碧云天公司购买检测转录因子NF-κB活性的报告基因质粒，同时用Renilla质粒作为内参进行双荧光素酶报告基因实验。在A549细胞中转染NF-κB-luc质粒和Renilla质粒，在过表达IKBKB后，检测IKBKB对NF-κB转录因子活性的影响。

### 1.8 数据统计

实验数据使用SPSS 17.0统计软件进行分析，Excel 2003作图。统计分析方法采用方差分析或*t*检验，以*P* < 0.05表示差异具有统计学意义。

## 2 结果

### 2.1 检测A549及A549/DDP细胞中*IKBKB*基因表达差异及两细胞株之间凋亡和药物敏感性差异

本实验应用real-time PCR技术检测*IKBKB*基因在两亲本细胞系中的mRNA表达水平差异，实验结果显示*IKBKB*基因在A549/DDP细胞株中高表达，表达量为A549细胞的1.9倍，差异具有统计学意义（*P* < 0.05）（图 1A）。应用MTT检测药物敏感性，A549及A549/DDP细胞在顺铂作用下的IC_50_值分别为（2.16±1.31）μg/mL、（14.32±1.18）μg/mL（*P* < 0.01）（图 1B）。为了检测两株细胞在顺铂作用下的凋亡差异，A549及A549/DDP细胞经浓度为2.5 μg/mL顺铂处理后，通过流式细胞仪检测显示凋亡率分别为（15.52±1.21）%、（7.62±0.82）%（*P* < 0.01）（图 1C），结果说明A549/DDP细胞耐药性和抗凋亡性均明显强于A549细胞。

**1:**
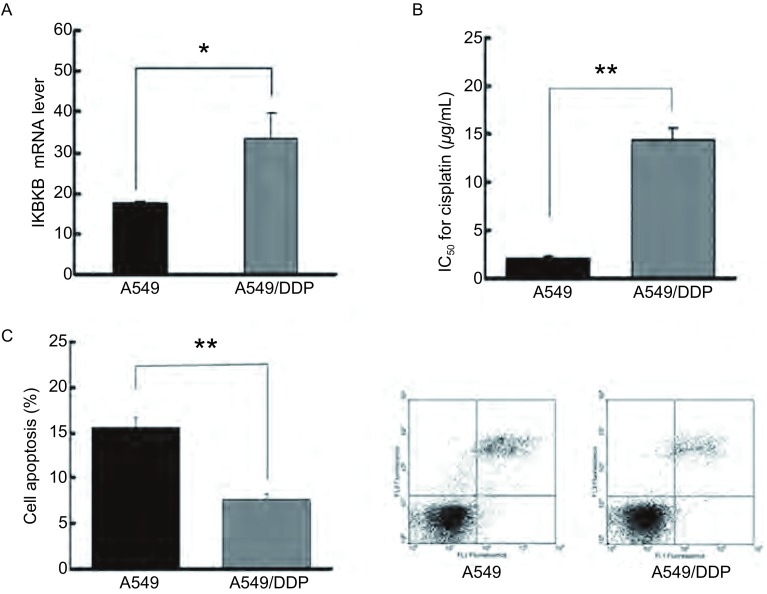
A549与A549/DDP细胞中*IKBKB*基因表达差异及两细胞株之间凋亡和药物敏感性差异。A：Real-time PCR检测*IKBKB*基因在A549与A549/DDP细胞株中的mRNA水平差异；B：MTT检测A549与A549/DDP细胞株顺铂的敏感性；C：流式细胞技术检测A549与A549/DDP细胞株的凋亡率；*P < 0.05；**P < 0.01. Detection of BRE expression and cisplatin-resistance in A549 and A549/DDP cells. A: Real-time PCR analysis of IKBKB expression in A549 and A549/DDP cells; B: MTT analysis of cell viability of A549 and A549/DDP cells after treated by cisplatin; C: Annexin V-FITC flow cytometry assay detects the apoptosis rate of A549 and A549/DDP cells after treated by cisplatin. *P < 0.05; **P < 0.01.

### 2.2 pcDNA3.1/IKBKB质粒构建及转染

pcDNA3.1/IKBKB质粒构建完成后进行酶切鉴定，琼脂糖电泳结果显示质粒被酶切成两个条带，分别对应marker为2, 000 bp及5, 000 bp（图 2），质粒测序显示碱基序列正确。与对照组（A549细胞转染pcDNA3.1空载体）和未处理的A549细胞相比，转染pcDNA3.1/IKBKB质粒的A549细胞中*IKBKB*基因在mRNA水平明显上升（图 3A），结果说明质粒构建及转染成功。

**2:**
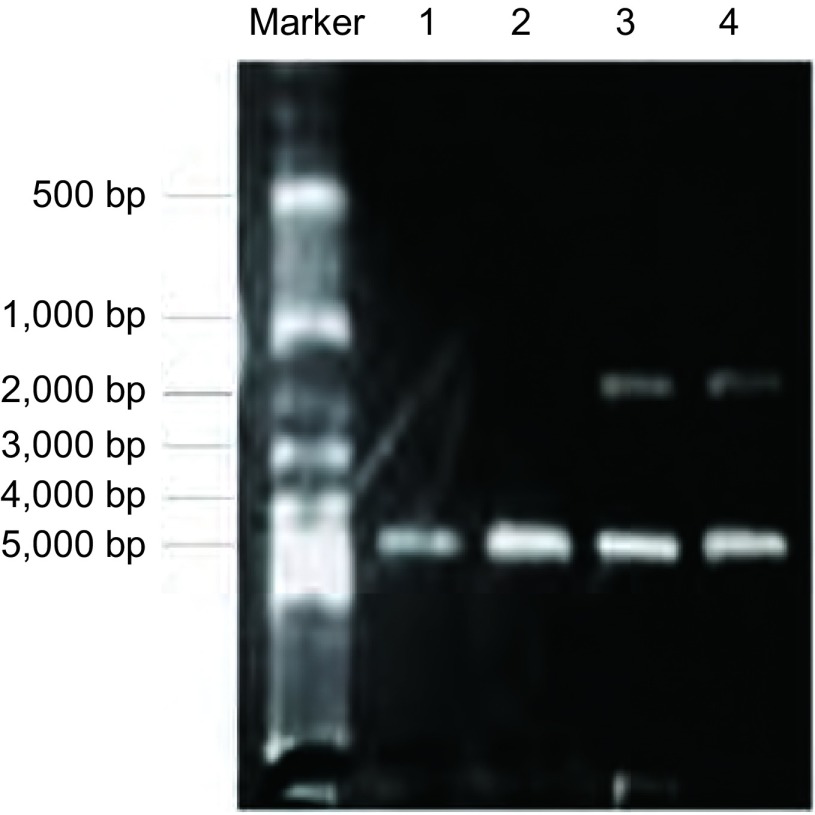
pcDNA3.1/IKBKB酶切产物的0.8%琼脂糖凝胶电泳图。1，2：pcDNA3.1/Hygro；3，4：pcDNA3.1/IKBKB经酶切后片段。 0.8% agrose gel electrophoresis of enzyme-digested products of pcDNA3.1/IKBKB; 1, 2: pcDNA3.1/Hygro; 3, 4: Enzyme-digested products of pcDNA3.1/IKBKB.

**3:**
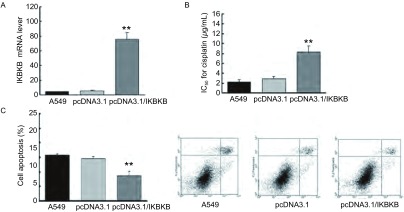
过表达*IKBKB*基因导致A549细胞顺铂耐药性增加。A：Real-time PCR检测*IKBKB*基因在转染pcDNA3.1/IKBKB质粒A549细胞、转染pcDNA3.1质粒A549细胞、未处理A549细胞中的差异；B：MTT检测转染pcDNA3.1/IKBKB质粒A549细胞、转染pcDNA3.1质粒A549细胞、未处理A549细胞对顺铂敏感性；C：流式细胞技术检测转染pcDNA3.1/IKBKB质粒A549细胞、转染pcDNA3.1质粒A549细胞、未处理A549细胞顺铂作用下的凋亡率；*P < 0.05; **P < 0.01. Up-regulation of IKBKB expression induces cisplatin-resistance in A549 cells. A: Real-time PCR analysis of *IKBKB* in A549 cells transfected with pcDNA3.1 or pcDNA3.1/IKBKB and untreated A549 cells; B: MTT analysis of cell viability of A549 cells transfected with pcDNA3.1 or pcDNA3.1/IKBKB and primary A549 cells after treated by cisplatin; C: Annexin V-FITC flow cytometry assay detects the apoptosis rate of A549 cells transfected with pcDNA3.1 or pcDNA3.1/IKBKB and primary A549 cells after treated by cisplatin. **P < 0.01.

### 2.3 *IKBKB*基因通过抑制细胞凋亡导致A549细胞顺铂耐药性增加

实验结果显示：转染pcDNA3.1/IKBKB质粒的A549细胞与对照组（A549细胞转染pcDNA3.1空载体）和未处理的A549细胞相比，IC_50_分别为（8.31±1.16）μg/mL、（2.91±0.98）μg/mL、（2.24±1.17）μg/mL（*P* < 0.01）（图 3B），结果表明过表达*IKBKB*基因的A549细胞耐药性明显增加；进一步通过流式细胞仪检测细胞凋亡情况，结果显示：转染pcDNA3.1/IKBKB质粒组与对照组（空载体组）和未处理组在顺铂作用下的凋亡率分别为（8.76±1.43）%、（14.69±2.01）%、(15.89±1.61)%（*P* < 0.01）（图 3C），结果提示*IKBKB*基因通过抑制细胞凋亡导致顺铂耐药。

### 2.4 pcDNA3.1/IKBKB质粒在A549细胞中的高表达激活NF-κB信号通路

本实验进一步研究了*IKBKB*基因导致A549细胞耐药的分子机制，我们对*IKBKB*基因下游的NF-κB信号通路进行研究。双荧光素酶报告基因实验显示（图 4），在A549细胞中过表达IKBKB后，NF-κB的活性高于转染对照空载体组，说明在肺腺癌细胞A549中，IKBKB确实可以激活下游NF-κB的转录活性。

**4:**
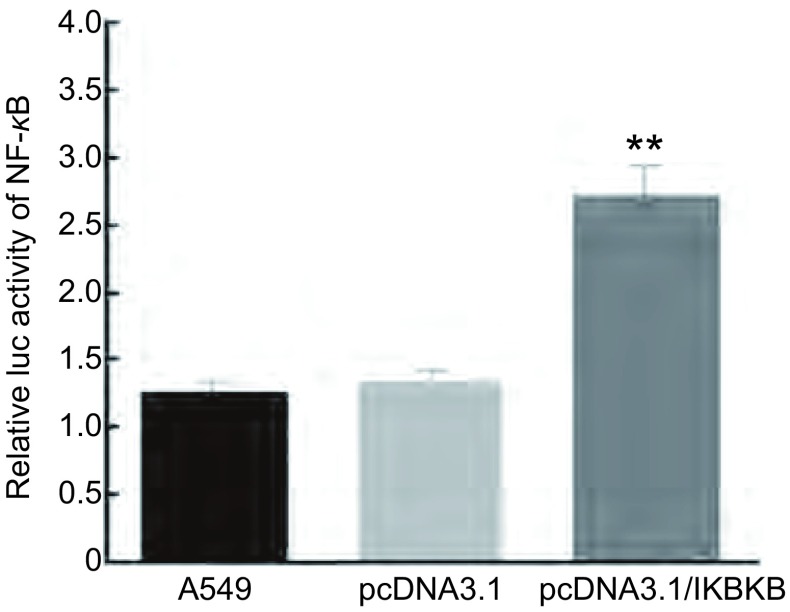
双荧光素酶报告基因检测显示：pcDNA3.1/IKBKB在A549细胞中激活NF-*κ*B的活性。*P < 0.05；**P < 0.01. Dual luciferase reporter gene experiment: In A549 cells, pcDNA3.1/IKBKB enhances the activity of NF-*κ*B. **P < 0.01.

## 3 讨论

顺铂耐药的产生涉及复杂分子机制，其中包括染色体异常、凋亡相关信号转导、凋亡抑制因子表达、DNA损伤修复基因表达、HER-2/neu高表达、PI3K/AKT信号通路的激活、P53功能缺失、caspase激活过程的阻断等^[5, 12]^，但是具体肿瘤细胞顺铂耐药性的产生机制尚不明确。有研究报道发现*IKBKB*基因在乳腺癌和头颈部鳞癌顺铂耐药产生中起重要作用^[10, 11]^，但是其在肺癌顺铂耐药研究中罕有报道。

IKBKB是组成IKK复合体的重要催化亚基之一，其在IKK复合体激活NF-κB过程中发挥重要作用^[9]^。为了研究*IKBKB*基因在肺癌顺铂耐药产生中的作用，我们应用real-time PCR技术检测发现*IKBKB*基因在肺腺癌A549/DDP细胞株中的表达明显高于A549细胞株，这说明*IKBKB*基因的高表达可能导致肺癌细胞顺铂耐药性的产生。实验结果显示，顺铂作用下的A549/DDP细胞株IC_50_明显高于A549细胞株，流式细胞技术检测发现A549/DDP细胞株凋亡率明显低于A549细胞。根据上述实验结果我们推测，IKBKB通过抑制肺腺癌细胞株凋亡过程导致耐药性增加。凋亡抑制在顺铂耐药性产生的机制中起重要作用^[3]^，为了明确*IKBKB*基因通过抑制凋亡导致肺癌顺铂耐药性产生的机制，我们通过在A549细胞中转染pcDNA3.1/IKBKB质粒过表达了*IKBKB*基因。实验结果显示IKBKB高表达的A549细胞耐药性明显增加，凋亡受到明显抑制，这与我们的提出的假设一致，说明IKBKB调控的凋亡抑制是肺癌产生顺铂耐药的机制之一。

有研究^[11]^报道，IKBKB在乳腺癌中通过抑制FOXO3导致顺铂耐药性产生。我们的研究发现，*IKBKB*基因的高表达激活了其下游的NF-κB信号通路。NF-κB是具有多向转录调节作用的核转录因子，其静息状态下位于细胞质中，激活后进入到细胞核内调控细胞凋亡、炎症、氧化应激等过程。NF-κB介导多种凋亡相关基因转录调控，具有抑制细胞凋亡的作用^[13]^，TNFR相关因子、JNK、Bcl-2家族等都参与了NF-κB激活后的抗凋亡过程^[14]^。有研究显示，NF-κB信号通路激活可以导致抗肿瘤药物耐药^[15]^，NF-κB信号通路的激活导致宫颈癌细胞产生顺铂耐药^[16]^，抑制NF-κB信号通路可以引发耐顺铂的胃癌细胞株（SGC7901/DDP）凋亡增加，逆转其耐药性^[17]^。我们的研究显示，*IKBKB*基因通过激活NF-κB信号通路抑制细胞凋亡。

本研究发现，*IKBKB*基因在细胞耐药性产生的机制中起着重要的调节作用。IKBKB通过激活NF-κB信号通路抑制细胞凋亡，从而使细胞产生耐药性。虽然具体机制有待进一步研究，但我们的研究丰富了肿瘤耐药性产生的机制，为新的抗肿瘤药物的研发提供了新靶点，对解决肿瘤耐药的难题有重要意义。
